# Dietary tryptophan mitigates lung ischemia-reperfusion injury in association with increased indole-3-propionate and aryl hydrocarbon receptor signaling

**DOI:** 10.1080/19490976.2026.2715839

**Published:** 2026-08-13

**Authors:** Tomohiro Chaki, Daisuke Maruyama, Thien N. M. Doan, Xiaoli Tian, Arun Prakash

**Affiliations:** a Department of Anesthesia and Perioperative Care, University of California San Francisco, San Francisco, CA, USA; b Department of Anesthesiology, Sapporo Medical University School of Medicine, Sapporo, Hokkaido, Japan; c Department of Anesthesia and Perioperative Care, San Francisco General Hospital, San Francisco, CA, USA

**Keywords:** Ischemia-reperfusion, dietary intervention, tryptophan, indole derivatives, indole-3-propionate, alveolar macrophage, aryl hydrocarbon receptor

## Abstract

**Background:**

Lung ischemia–reperfusion (IR) injury drives early morbidity after lung transplantation and cardiothoracic surgery, yet targeted preventive therapies are lacking. The gut–lung axis and microbiota-derived tryptophan metabolites, including indole-3-propionate (IPA), may regulate pulmonary immunity and inflammation. We investigated whether a tryptophan-rich (Trp-Rich) diet attenuates sterile lung IR injury by increasing microbiota-derived indole metabolites and reprogramming alveolar macrophage (AM) inflammatory responses.

**Methods:**

C57BL/6 mice receiving isocaloric tryptophan-standard (Trp-Std) or tryptophan-rich (Trp-Rich) diets underwent lung IR injury. Oxygen saturation, lung cytokines, and aryl hydrocarbon receptor (AhR) signaling readouts were evaluated. Gut microbiota was profiled by 16S rRNA sequencing, and targeted metabolomics quantified tryptophan metabolites in feces, portal vein (PV) plasma, and lung tissue. To further assess inflammatory priming *in vivo*, mice were additionally challenged with intratracheal lipopolysaccharide (LPS). Mechanistic studies compared IPA with related indoles in cell lines and primary human AMs, including *ex vivo* nutritional IR, LPS stimulation, and AhR stimulation and blockade using synthetic agonists and antagonists.

**Results:**

Trp-Rich feeding improved post-IR oxygenation, reduced lung IL-1β, and increased pulmonary AhR-downstream gene expression. Trp-Rich diet remodeled gut microbiota, enriching for *Bifidobacterium* and *Lactobacillus*, and increasing IPA levels across feces, PV plasma, and lung tissue. In the LPS intratracheal challenge, Trp-Rich feeding reduced IL-6 levels in lung tissue and systemic plasma. Primary AMs isolated from Trp-Rich mice also showed reduced IL-1β and IL-6 release in an *ex vivo* nutritional IR model. Among the tested indole metabolites, IPA showed the strongest dose-dependent suppression of LPS-induced cytokines and chemokines, suppressed *ex vivo* nutritional IR injury, and its effects were attenuated by pharmacologic AhR blockade.

**Conclusions:**

A Trp-Rich diet attenuated sterile lung IR injury, coinciding with gut microbiota remodeling, increased systemic and pulmonary IPA, reduced inflammatory priming, and reprogrammed AM responses. These data support diet- or microbiome-directed strategies targeting IPA–AhR signaling to mitigate perioperative lung IR injury.

## Introduction

Lung ischemia-reperfusion (IR) injury constitutes a significant contributor to early morbidity and mortality following lung transplantation and cardiothoracic surgical procedures, characterized by an abrupt onset of sterile inflammation, disruption of endothelial and epithelial barriers, and neutrophil-predominant alveolitis.^1-3^ Recent experimental and translational investigations have elucidated the pivotal roles of activated alveolar macrophages (AMs) in exacerbating vascular permeability and diffuse alveolar damage.^4-8^ Mechanistically, lung IR injury is consistent with a two-signal inflammatory theory in which endogenous damage-associated molecular patterns (DAMPs) and TLR4-related inflammatory priming contribute to downstream cytokine production, while IR-associated stressors can promote inflammasome activation, including NOD-like receptor family pyrin domain containing 3 (NLRP3) activation.^8-11^ Nevertheless, no specific therapeutic interventions for either signal have been incorporated into routine clinical practice. These limitations underscore the need for innovative strategies that modulate upstream inflammatory pathways rather than solely addressing downstream injury.

Compelling evidence positions the gut–lung axis as a fundamental regulator of pulmonary immune responses.^12^ Alterations in both the composition and functionality of the intestinal microbiota significantly influence susceptibility to pneumonia, acute respiratory distress syndrome (ARDS), and chronic pulmonary diseases through microbially derived metabolites, such as short-chain fatty acids, tryptophan derivatives, and others, which orchestrate myeloid and epithelial responses at remote mucosal locations.^13-18^ Mechanistic investigations reveal that gut-derived metabolites can modulate myeloid and tissue-resident immune cell responses at distant sites.^12^ ^,^ ^19^ ^,^ ^20^ Conversely, dysbiosis or the depletion of beneficial microbes and metabolites aggravates lung inflammation. These findings suggest that salutary dietary or microbiota-based interventions may provide an indirect protective effect against lung IR injury by reprogramming systemic and pulmonary immune responses.^21^ Nonetheless, this concept remains inadequately explored within the context of acute sterile lung injury.

Among microbial metabolites, indole derivatives generated from dietary tryptophan are emerging as key regulators of mucosal immunity.^22^ Indole-3-propionate (IPA) is a prominent member of the microbiota-derived indole family, which also includes indole-3-acetate (IAA) and indole-3-lactate (ILA). IPA exerts antioxidant and anti-inflammatory effects across experimental models, including suppression of NF-κB and inflammasome signaling, preservation of barrier integrity, and modulation of macrophage phenotypes via aryl hydrocarbon receptor (AhR) signaling.^23-25^ Microbiota-derived indoles can act through AhR-dependent pathways to regulate immune responses at barrier and distant tissues.^19^ ^,^ ^20^ Microbiota-derived indoles have also been linked to protection against systemic inflammation and mitigation of mucosal and pulmonary injury in infection-related models.^26^ ^,^ ^27^ However, it remains unclear whether a dietary strategy that increases microbial indole metabolism can raise endogenous IPA across the gut and circulation, particularly within the lung, and whether these changes are coupled with reduced lung inflammation and altered AhR-related responses during sterile lung IR injury.

Consequently, we posited that a diet rich in tryptophan would modify gut microbiota composition and enhance the production of gut-derived tryptophan metabolites, particularly IPA, reducing lung inflammation following IR injury. Utilizing a murine lung IR model in conjunction with targeted metabolomics, gut microbiota profiling, and *in vitro* assays with MH-S and human AMs, we sought to elucidate the gut–lung axis that underlies the protective effects mediated by tryptophan metabolites in acute lung IR injury.

## Materials and methods

Methods are summarized here. Full experimental procedures and analysis parameters are provided in the Supplemental Methods.

### Animal care

All animal procedures were approved by the Institutional Animal Care and Use Committee at the University of California San Francisco (UCSF) (protocol: AN197325-01). Wild-type C57BL/6 mice (10–15 weeks old, both sexes) were housed under standard conditions with free access to food and water. Mice used for the lung IR experiment were bred and maintained within the UCSF Laboratory Animal Resource Center (LARC), whereas mice used for the intratracheal lipopolysaccharide (LPS) challenge and primary murine alveolar macrophage experiments were purchased from The Jackson Laboratory. The mouse source was not mixed within each experiment. Mice purchased from The Jackson Laboratory were acclimated for at least 2 weeks after arrival before dietary intervention, and LARC-bred mice were maintained under standard facility conditions for at least 2 weeks before dietary intervention. Mice were group-housed according to UCSF LARC guidelines, with 2–5 mice per cage, and single housing was not used. Because diets were administered at the cage level, mice were housed by diet group. Individual mice were analyzed as biological replicates, while acknowledging that diet exposure occurred at the cage level. Mice were randomized to an isocaloric, isonitrogenous tryptophan-standard diet (Trp-Std, 0.18%) or tryptophan-rich diet (Trp-Rich, 0.60%) for 14 d (Inotiv). Group sizes were Trp-Std (males *n* = 14, females *n* = 13) and Trp-Rich (males *n* = 12, females *n* = 11) in the lung IR experiment, Trp-Std (males *n* = 10) and Trp-Rich (males *n* = 8) in the intratracheal LPS challenge experiment, and Trp-Std (males *n* = 10) and Trp-Rich (males *n* = 10) in the primary AMs experiment. Cellulose content was matched between diets (3%) to minimize fiber-driven microbiome differences (Supplemental Table S1). Of note, body weight did not differ between groups (Figure S1A), and gross intestinal appearance was comparable after 2 weeks of dietary intervention (Figure S1B). Complete blinding during diet administration and lung IR surgery was not feasible because diets were administered at the cage level, and the surgeon was involved in cage and diet management. To minimize observer bias where possible, SpO₂ measurement and lung sample collection were performed with investigators blinded to diet-group assignment. Samples submitted for targeted metabolomics and 16S rRNA sequencing were coded before measurement and sequencing. ELISA and qPCR assays were performed without blinding. Final statistical analyses were performed after decoding group labels.

### Left lung IR surgery and sample collection

Unilateral left lung IR was performed as previously described.^8^ ^,^ ^28^ ^,^ ^29^ In brief, mice were anesthetized, intubated, and mechanically ventilated, and the left pulmonary artery was occluded using a reversible slipknot for 60 min, followed by 60 min of reperfusion. Peripheral oxygen saturation and respiratory rate were measured immediately prior to euthanasia (MouseOx Plus) and assessed in male mice only, as the MouseOx neck-collar sensor is recommended for animals >25 g, and its use in smaller female mice could compromise breathing. Blood was collected from the inferior vena cava (IVC) and portal vein (PV) into heparinized syringes, centrifuged, and the plasma was stored at −80 °C. The left lung was divided for RNA extraction and cytokine quantification, and the right lung was used for targeted metabolomics. Fecal pellets were collected from the colon and stored at −80 °C for 16S rRNA sequencing and metabolomics. Lung IR surgeries were performed over multiple experimental days because a maximum of five mice could be processed per day. Because diets were administered at the cage level and mice were group-housed by diet group, surgical scheduling was organized by cage and diet group. Some experimental days included mice from both diet groups, whereas others included mice from a single diet group. Surgical days were scheduled in a non-systematic order to avoid completing all procedures for one diet group before the other. Sample collection, tissue processing, and storage procedures were kept consistent across experimental days.

### Intratracheal LPS challenge

To evaluate whether dietary tryptophan enrichment alters pulmonary inflammatory responsiveness in a standardized PAMP-driven challenge, a subset of mice after 2 weeks of dietary intervention received intratracheal LPS (5 mg/kg). Lung tissue and IVC plasma were collected 6 h later for cytokine quantification. The intratracheal LPS challenge was performed over multiple experimental batches using the same LPS dose and the same 6-h collection interval across groups.

### Reagents and cell lines

IPA, ILA, IAA, indole, L-tryptophan, LPS (*E. coli* 0111:B4), and the AhR antagonist CH-223191 were purchased from Sigma–Aldrich. TCDD was purchased from Neta Scientific. MH-S cells (murine AM line) were obtained from ATCC and cultured in RPMI 1640 supplemented with 10% fetal bovine serum (FBS) and 1% penicillin-streptomycin.

### Collection of human primary AMs

Primary human AMs were obtained by bronchoalveolar lavage from adult donor lungs declined for transplantation (UCSF, provided by Dr. Michael Matthay). Cells were processed to remove erythrocytes, resuspended in RPMI 1640 with 10% FBS and antibiotics, and allowed to adhere before stimulation experiments. Donor information is summarized in Supplemental Table S2.

### *In vitro and ex vivo* AM assays

For MH-S experiments, cells were pretreated with tryptophan-related metabolites (IPA, ILA, IAA, indole, or L-tryptophan; 0.01–1 mM) and then co-treated with LPS (50 ng/mL) in the continued presence of the metabolites. For AhR-dependence experiments, cells were pretreated with CH-223191 and then exposed to IPA (0.01–0.1 mM) or TCDD (10 nM), followed by LPS co-treatment, with or without CH-223191. For human AM experiments, cells were co-treated with LPS (10 ng/mL) and tryptophan-related metabolites (0.01–1 mM) overnight.

*Ex vivo* nutritional IR using human and mouse primary AMs was performed as previously described.^21^ ^,^ ^28^ Briefly, we co-treated primary AMs with LPS (200 ng/mL) and IPA overnight, washed, incubated in phosphate-buffered saline (PBS) for 1 h (ischemia-mimicking phase), and then switched to RPMI 1640 medium (reperfusion-mimicking phase). Supernatants were collected for cytokine measurements, and in the human AM experiment, cell number and confluency were quantified by imaging for normalization.

### Cytokine measurement and gene expression

Mouse lung homogenates and cell-culture supernatants were analyzed using DuoSet ELISA kits (R&D Systems). For *in vivo* comparisons, cytokine concentrations measured by ELISA were analyzed on the same assay plate to minimize inter-assay variability. Total RNA was extracted from lung tissue or cells, reverse-transcribed, and analyzed by RT-qPCR using TaqMan assays. Gene-expression analyses were performed across multiple qPCR plates when not all samples could be accommodated on a single plate. However, samples from both Trp-Std and Trp-Rich mice were included on each qPCR plate to minimize plate-specific bias between diet groups. Relative gene expression was calculated using the 2^-ΔΔCt method with *Actb*, *Gapdh*, and *Rpl19* as reference genes.

## 16S rRNA gene sequencing and microbiome analysis

For unbiased microbiome analysis, we analyzed the first four mice enrolled per group (*n* = 4 per group). Microbial DNA was extracted from fecal samples (QIAamp PowerFecal Pro DNA Kit, Qiagen). The V4 region of the bacterial 16S rRNA gene was amplified using the 515F/806R primer pair, pooled, purified, and sequenced on an Illumina MiSeq platform (2 × 300 bp). Demultiplexed paired-end reads were subsequently processed using a DADA2-based workflow in MicrobiomeAnalyst, and taxonomy was assigned against SILVA v138.^30^ ^,^ ^31^

### Targeted metabolomics of tryptophan-related metabolites

Tryptophan-related metabolites were quantified in feces, PV plasma, and lung using targeted LC-MS with isotope-labeled internal standards and external calibration performed by the metabolomics core at the University of Michigan (Maureen Kachmann). Samples were extracted using matrix-specific solvent protocols, analyzed with pooled quality-control injections, and quantified using MassHunter software. Tissue concentrations were normalized to input mass.

### Multi-omics integration and statistical analysis

Microbiome profiles were generated in MicrobiomeAnalyst using a DADA2-based ASV workflow with standard feature filtering.^30^ ^,^ ^31^ *β*-diversity was evaluated using Bray-Curtis and UniFrac distances and tested by PERMANOVA. Differential abundance was assessed using DESeq2 with FDR correction, and predicted functional potential was inferred using Tax4Fun2 (KEGG ortholog profiles). Targeted metabolomics data were analyzed in MetaboAnalyst 6.0 with log transformation and scaling, with group comparisons performed using FDR-adjusted univariate testing and visualization by ordination and volcano plots.^32^ ^,^ ^33^ Associations between taxa, metabolites, and lung inflammatory/AhR readouts were assessed using Spearman correlations.

All other statistical analyses were performed in GraphPad Prism 10 (GraphPad Software, Boston, MA). Data are presented as mean ± SEM. Two-group comparisons used unpaired t-tests, or Mann-Whitney tests as appropriate. Multi-group comparisons were performed using one-way ANOVA with Tukey’s post hoc test. A two-sided *p*-value < 0.05 was considered significant.

## Results

### A Trp-Rich diet attenuates lung IR injury and activates AhR signaling

**Figure 1.**
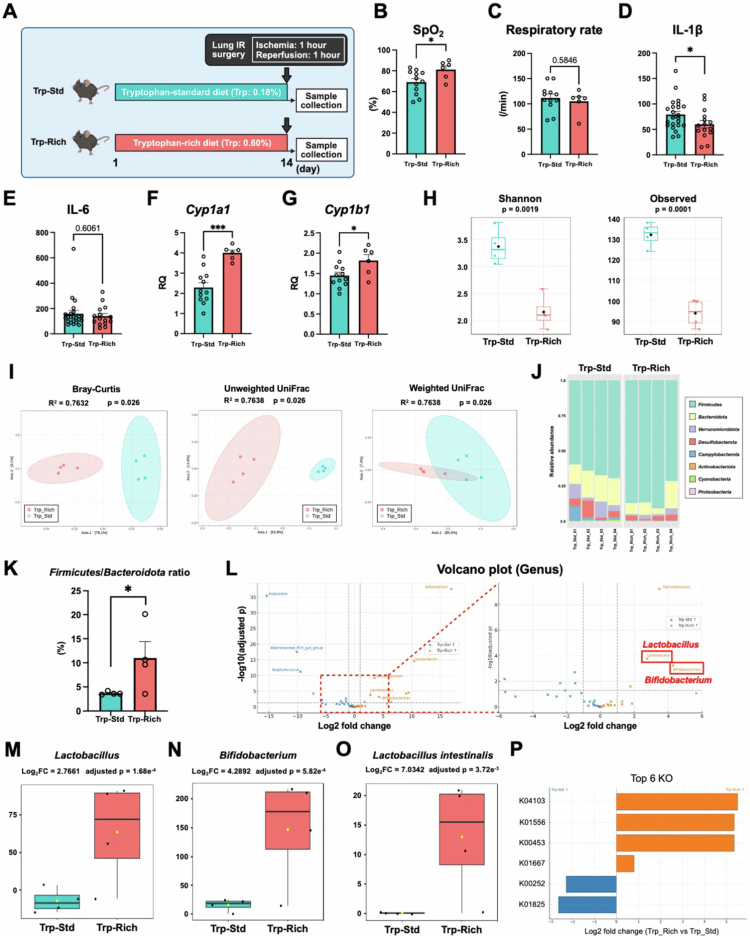
Tryptophan-rich diet attenuates lung ischemia-reperfusion injury and remodels the gut microbiota. (A) Study design. Mice were assigned to isocaloric tryptophan-standard (Trp-Std, 0.18% tryptophan) or tryptophan-rich (Trp-Rich, 0.60% tryptophan) diets for 2 weeks, followed by unilateral lung ischemia-reperfusion (60 min ischemia and 60 min reperfusion) and sample collection. (B, C) Oxygen saturation (SpO_2_) and respiratory rate were measured after 60 min of reperfusion. SpO_2_ was higher in Trp-Rich mice, whereas the respiratory rate did not differ between groups (Trp-Std, *n* = 12; Trp-Rich, *n* = 6). (D, E) Lung inflammatory cytokines quantified by ELISA showing reduced IL-1β in the Trp-Rich group, with no significant difference in IL-6 (Trp-Std, *n* = 23; Trp-Rich, *n* = 15). (F, G) RT-qPCR analysis of aryl hydrocarbon receptor (AhR) target genes (*Cyp1a1* and *Cyp1b1*) demonstrating increased expression in Trp-Rich lungs (Trp-Std, *n* = 12; Trp-Rich, *n* = 6). (H) Alpha-diversity metrics (Shannon index and observed features) showing reduced diversity in the Trp-Rich group. (I) *β*-diversity ordinations based on Bray-Curtis, unweighted, and weighted UniFrac distances demonstrating separation of Trp-Std and Trp-Rich communities (PERMANOVA). (J) *Phylum*-level relative abundance and (K) *Firmicutes*/*Bacteroidota* ratio, which was increased by the Trp-Rich diet. (L) *Genus-*level differential abundance volcano plot highlighting taxa altered by diet. (M–O) Relative abundances of *Lactobacillus*, *Bifidobacterium*, and *Lactobacillus intestinalis*, each enriched in Trp-Rich mice. The taxonomy chart at the *genus* level and volcano plot at the *species* level are presented in Supplemental Figure S2. (P) Predicted functional profiling of the tryptophan metabolism pathway (ko00380) showing enrichment of predicted KEGG orthologs related to indole-kynurenine metabolism in the Trp-Rich group (K04103, K01556, K00453, and K01667). PERMANOVA was used for *β*-diversity comparisons, and other comparisons were performed using an unpaired t-test or the Mann‒Whitney test with false discovery rate correction for multiple testing. *n* = 4 in each group for microbiome analysis. *: *p* < 0.05, ***: *p* < 0.001.

To assess the *in vivo* impact of dietary tryptophan, mice were fed a Trp-Rich or Trp-Std diet for 2 weeks (Figure 1A). After left lung IR, oxygen saturation was higher in the Trp-Rich group despite similar respiratory rates, indicating improved lung function (Figure 1B, C). In left lung homogenates, IL-1β was reduced in the Trp-Rich group, whereas IL-6 did not differ between groups (Figure 1D, E). Given prior evidence that estrogen can mitigate lung IR injury,^34^ we examined sex-specific responses. The protective phenotype was not observed in females but was preserved in males (Figures S1C, D). Therefore, subsequent analyses were performed in male mice, and the sex-dependent effects of dietary tryptophan on lung injury responses warrant further study, in keeping with prior reports that lung IR injury is modulated by sex and estrogen signaling.^34^ Lung expression of the canonical AhR target genes *Cyp1a1* and *Cyp1b1* was increased in the Trp-Rich group, indicating successful enhancement of AhR signaling (Figure 1F, G).^35^ ^,^ ^36^ Given the absence of a clear protective phenotype in females, we next performed additional RT-qPCR analyses of *Ahr*, *Cyp1a1*, and *Cyp1b1* in injured lung tissue from female mice after IR and compared these findings with the corresponding male data (Figure S1E–G). In female mice, Trp-Rich feeding significantly increased *Cyp1a1* expression, whereas *Cyp1b1* and *Ahr* expression were not significantly altered. When male and female data were examined together, *Cyp1a1* expression differed by both diet and sex, and the Trp-Rich-associated *Cyp1a1* response appeared lower in females than in males (Figure S1E). These findings suggest that Trp-Rich feeding activates at least part of the AhR-related transcriptional response in females, although the pattern of AhR target-gene expression was not identical between males and females.

### Remodeling of gut microbiota by a Trp-Rich diet

Using fecal 16S rRNA sequencing, we evaluated how a Trp-Rich diet alters gut microbial communities. Alpha-diversity was lower in the Trp-Rich group compared with the Trp-Std group (Figure 1H and S2A–D). Beta-diversity analyses also demonstrated clear group separation across Bray-Curtis, unweighted UniFrac, and weighted UniFrac distances (Figure 1I). At the *phylum* level, the Trp-Rich diet increased *Firmicutes* and decreased *Campylobacterota* and *Cyanobacteria*, resulting in a higher *Firmicutes*/*Bacteroidota* ratio (Figures 1J–K, Supplemental Table S3). At the *genus* level, *Faecalibaculum* and *Dubosiella* were dominant taxa (Figure S2E). Differential abundance analysis identified several taxa enriched in the Trp-Rich group at the *genus* level, including *Lactobacillus* and *Bifidobacterium*, whereas *Dubosiella*, *Rikenellaceae_RC9_gut_group*, and *Staphylococcus* were relatively depleted (Figure 1L, Supplemental Table S4). At the putative *species* level based on 16S V4 taxonomic assignment, *Lactobacillus intestinalis*, *Ileibacterium valens*, and *Faecalibaculum rodentium* were enriched in the Trp-Rich group, while *Streptococcus danieliae and Helicobacter ganmani* were depleted (Figure S2F, Supplemental Table S5). The expansion of *Lactobacillus*, *Bifidobacterium*, and *Lactobacillus intestinalis* in Trp-Rich mice (Figures 1M–O) was observed, and these taxa have been linked to increased production of indole-related tryptophan metabolites, including ILA and IPA, in the gut.^37^ ^,^ ^38^ Consistent with these taxonomic shifts, predicted functional profiling (Tax4Fun2, SILVA) followed by metagenomeSeq analysis indicated higher predicted abundance of selected KEGG orthologs in the tryptophan metabolism pathway in the Trp-Rich group, including K04103 (ipdC), K01667 (tnaA), and K00453 (TDO/TDO2) within the tryptophan metabolism pathway (ko00380). These findings suggest diet-associated changes in predicted community-level tryptophan metabolic potential, although they do not establish the microbial origin of the observed increase in IPA (Figure 1P).

### Transmission of tryptophan metabolites along the gut–lung axis with a Trp-Rich diet

**Figure 2.**
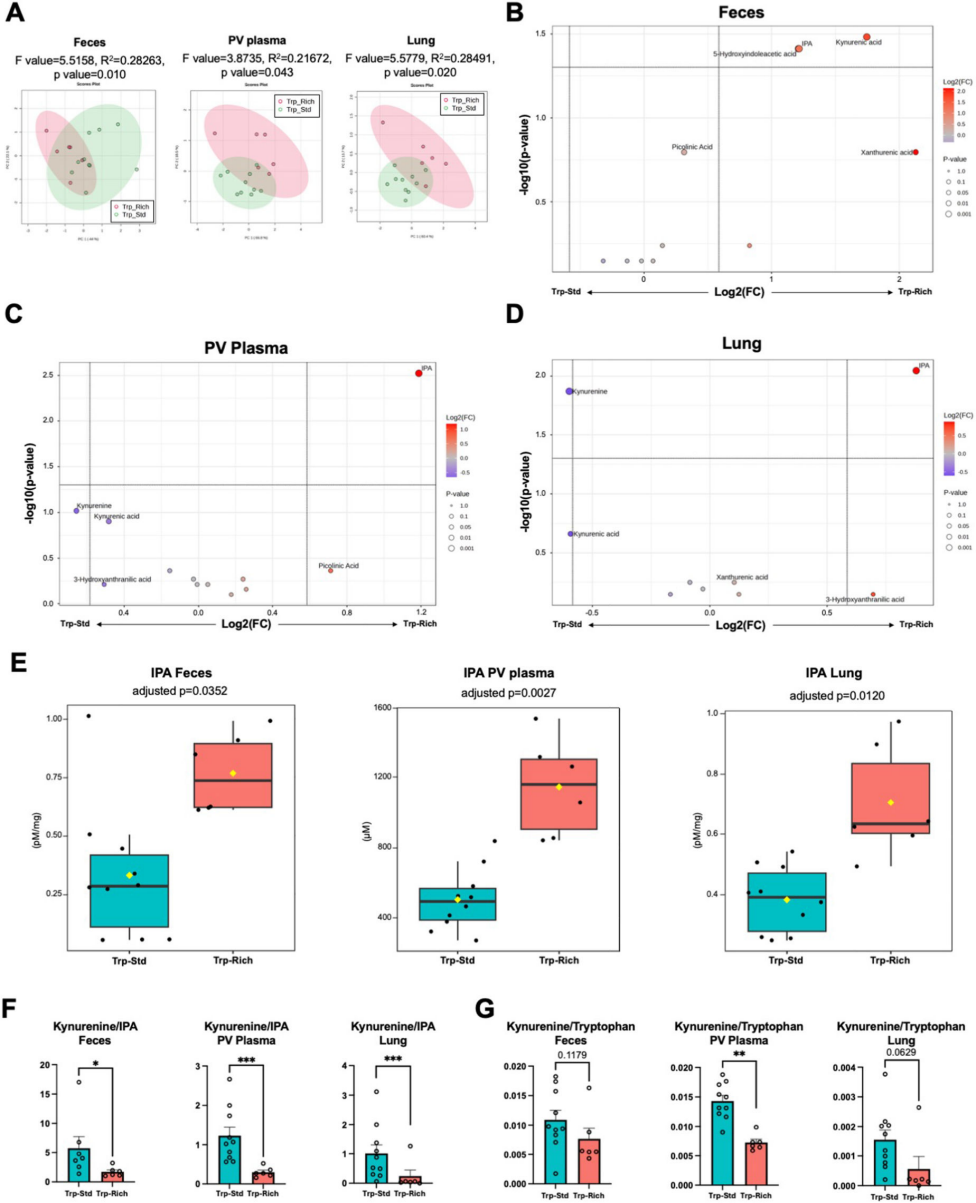
A tryptophan-rich diet reshapes tryptophan metabolism. (A) Principal component analysis (PCA) of targeted tryptophan metabolite profiles in feces, portal vein (PV) plasma, and lung tissue, showing separation of tryptophan-standard (Trp-Std) and tryptophan-rich (Trp-Rich) groups in each matrix. (B–D) Volcano plot demonstrating differential abundance of targeted tryptophan metabolites in feces, PV plasma, and lung tissue between Trp-Std and Trp-Rich groups. The complete panels of targeted tryptophan metabolites are shown in Supplemental Figure S3. (E) Indole-3-propionate (IPA) concentrations in the feces, PV plasma, and lung, demonstrating increased IPA in Trp-Rich mice across all three matrices. (F) Kynurenine/IPA ratios in feces, PV plasma, and lung, showing reduced ratios in the Trp-Rich group. (G) Kynurenine/tryptophan ratios in feces, PV plasma, and lung, with a significant reduction in PV plasma in Trp-Rich mice. Together, these ratio-based metrics support a relative shift in pathway balance toward indole/IPA production with Trp-Rich feeding as indirect surrogates rather than direct flux measurements. PERMANOVA evaluated group separation in PCA space, and other comparisons were performed using an unpaired t-test or the Mann–Whitney test with false discovery rate correction for multiple testing. Trp-Std, *n* = 10; Trp-Rich, *n* = 6. *: *p* < 0.05, **: *p* < 0.01, ***: *p* < 0.001.

Targeted metabolomics of tryptophan-related metabolites in feces, PV plasma, and right lung was performed to assess how a Trp-Rich diet reshapes tryptophan metabolic profiles. Principal component analysis demonstrated distinct metabolic profiles between Trp-Std and Trp-Rich groups across all matrices (Figure 2A). Volcano plots showed that IPA was consistently increased in the Trp-Rich group in feces, PV plasma, and lung (Figure 2B–E). In addition, kynurenic acid and 5-hydroxyindoleacetic acid were increased in feces in the Trp-Rich group, whereas kynurenine in the lung was increased in the Trp-Std group. To capture pathway-level balance, we calculated kynurenine/IPA and kynurenine/tryptophan ratios.^39^ ^,^ ^40^ The kynurenine/IPA ratio was lower in feces, PV plasma, and lung in the Trp-Rich group (Figure 2F). The kynurenine/tryptophan ratio was also reduced in PV plasma, with no differences in feces or lung (Figure 2G). In contrast, ILA and IAA did not differ between groups in any matrix (Figure S3). Together, these data indicate that Trp-Rich feeding was associated with a shift in tryptophan metabolite profiles toward higher IPA relative to kynurenine across gut, portal, and lung compartments.

### Integrated multi-omics analyses

**Figure 3.**
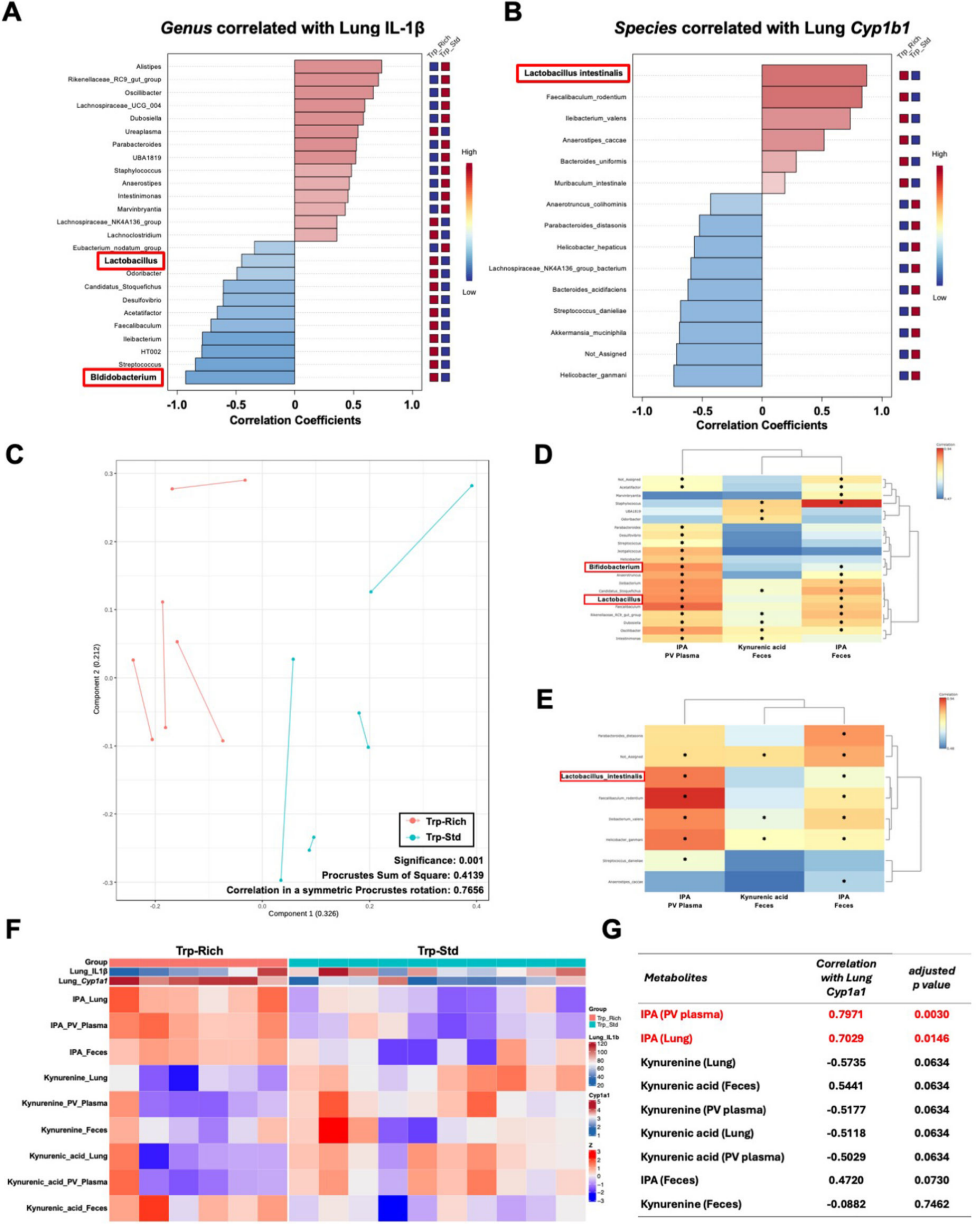
Integrated multi-omics analyses among gut microbiome, tryptophan metabolism, and lung inflammatory/aryl hydrocarbon receptor signaling readouts. (A) *Genus*-level correlations between the gut microbiota and lung inflammatory markers showed that *Bifidobacterium* abundance was inversely correlated with lung IL-1β. (B) *Species*-level correlations showed that *Lactobacillus intestinalis* abundance was positively correlated with lung *Cyp1b1* expression. Additional correlation analyses are provided in Supplemental Figure S4. (C) Procrustes analysis comparing microbiome and metabolomics ordinations, indicating overall concordance between datasets based on generally short Procrustes distances. (D, E) Correlation screening/heatmap analysis linking gut taxa with key metabolites, highlighting positive associations of *Bifidobacterium*, *Lactobacillus*, and *Lactobacillus intestinalis* with indole-3-propionate (IPA) in PV plasma. (F, G) Correlation heatmap/plots linking lung *Cyp1a1* expression with IPA levels in PV plasma and lung. Associations were assessed using Spearman’s rank correlation with false discovery rate correction. Trp-Std, *n* = 10; Trp-Rich, *n* = 6. In microbiome-related analysis, *n* = 4 in each group. *: *p* < 0.05.

To link diet-induced changes across the microbiome, metabolome, and host lung readouts, multi-omics correlation analyses were performed.^30-32^ ^,^ ^41^ At the *genus* level, *Bifidobacterium* was inversely correlated with lung IL-1β and IL-6 (Figure 3A and S4A, B), whereas correlations between *Lactobacillus* and these cytokines were not significant (Figure 3A, S4A, and S4C). At the *species* level, *Lactobacillus intestinalis* showed an inverse correlation with lung IL-6 (Figures S4D-E). Vis-à-vis AhR signaling, *Lactobacillus intestinalis* was positively correlated with lung *Cyp1b1* (Figure 3B and S4E); however, correlations involving *Bifidobacterium* or *Lactobacillus* with *Cyp1a1* or *Cyp1b1* were not significant (Figure S4B, C).

Procrustes analysis indicated overall concordance between microbiome and metabolomics ordinations, with a subset of samples showing larger discrepancies (Figure 3C). Correlation analysis further identified significant associations between *Bifidobacterium* and *Lactobacillus* and PV plasma IPA at the *genus* level (Figures 3D and S4F) and between *Lactobacillus intestinalis* and PV plasma IPA at the *species* level (Figures 3E and S4F). Finally, PV plasma and lung IPA levels were positively correlated with lung *Cyp1a1* (Figures 3F, G and S4G-H). Collectively, these integrated analyses support a model in which Trp-Rich feeding is associated with gut microbiota remodeling, increased IPA across multiple compartments, and AhR-related lung responses linked to a reduced inflammatory phenotype after IR injury. However, these association-based analyses do not by themselves establish microbial causality for IPA production.

### A Trp-Rich diet suppresses LPS-induced IL-6 responses *in vivo*

**Figure 4.**
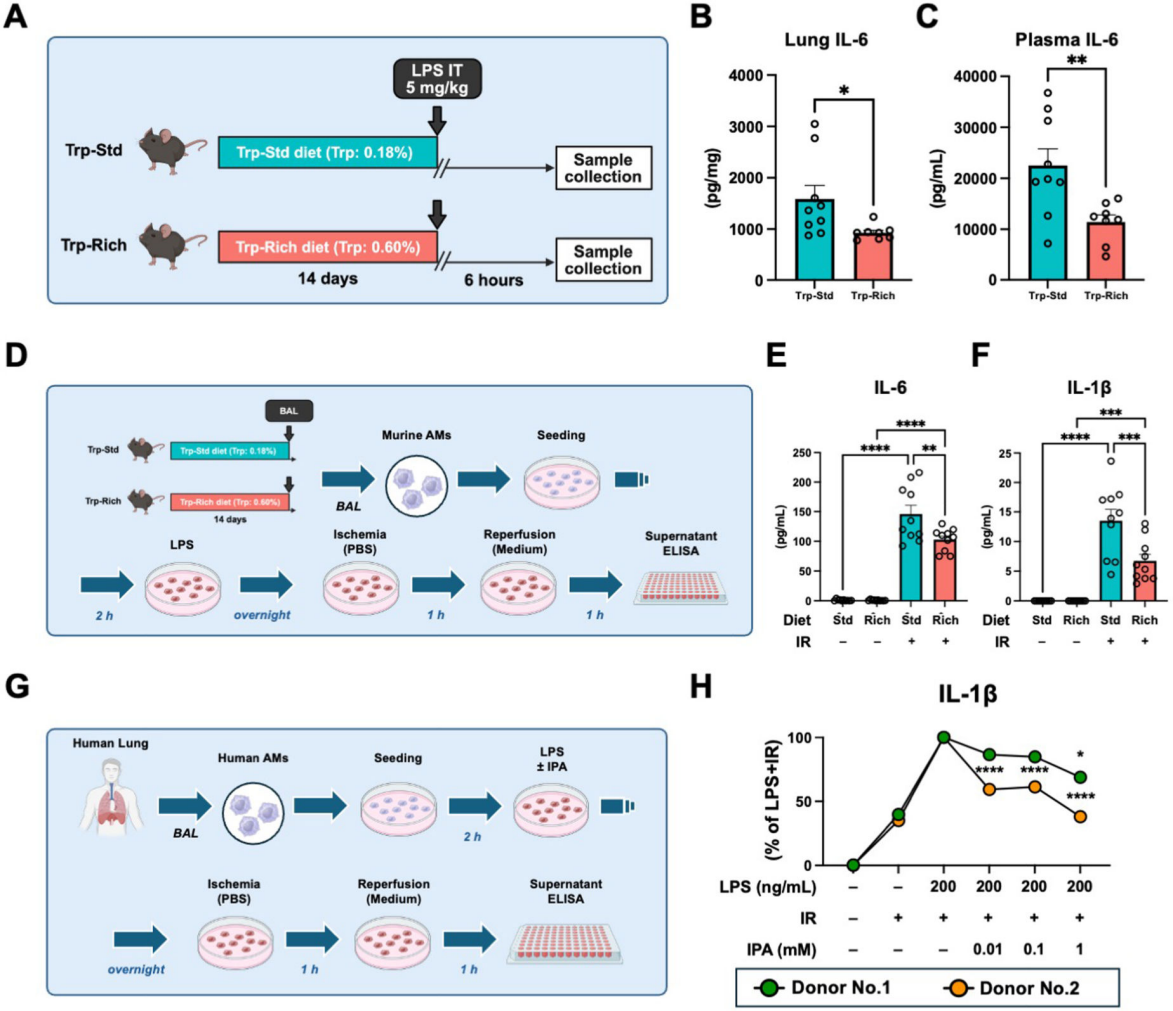
A tryptophan-rich diet attenuates lipopolysaccharide-induced inflammatory responsiveness *in vivo* and reprograms murine and human alveolar macrophage responses *ex vivo*. (A) Experimental design for the lipopolysaccharide (LPS) intratracheal (IT) challenge. Mice were fed isocaloric tryptophan-standard (Trp-Std, 0.18% tryptophan) or tryptophan-rich (Trp-Rich, 0.60% tryptophan) diets for 2 weeks, followed by LPS IT administration and sample collection 6 h later. (B, C) A Trp-Rich diet significantly reduced IL-6 levels in systemic plasma and lung tissue after LPS challenge. (D) Schematic of the *ex vivo* nutritional ischemia-reperfusion (IR) model using primary murine alveolar macrophages (AMs) from mice fed the Trp-Std or Trp-Rich diet for 2 weeks. (E, F) A Trp-Rich diet significantly reduced IL-6 and IL-1β production in the supernatant after nutritional IR. (G) Schematic of the *ex vivo* nutritional ischemia-reperfusion model using primary human AMs. (H) Indole-3-propionate (IPA) reduced IL-1β production in primary human AMs in a dose-dependent manner in two donor lungs. Data in B–C were analyzed by the Mann–Whitney U test (Trp-Std, *n* = 9; Trp-Rich, *n* = 8). Data in E-F were analyzed by one-way ANOVA with Tukey’s post hoc test (Trp-Std, *n* = 10; Trp-Rich, *n* = 10). Data in H were analyzed by one-way ANOVA with Tukey’s post hoc test. *: *p* < 0.05, **: *p* < 0.01, ***: *p* < 0.001, ****: *p* < 0.0001 in (B–C and E–F), *: *p* < 0.05 and ****: *p* < 0.0001 vs LPS + IR in (H).

To determine whether Trp-Rich feeding alters pulmonary inflammatory responsiveness in a standardized PAMP-driven setting, we performed an intratracheal LPS challenge (5 mg/kg) and quantified IL-6 in lung tissue and IVC plasma at 6 h (Figure 4A). Lung and plasma IL-6 levels were significantly lower in Trp-Rich mice, indicating attenuated pulmonary and systemic cytokine responses to LPS (Figure 4B, C). Consistent with reduced IL-6 protein, lung *Il6* transcript levels were also lower at 6 h after intratracheal LPS challenge (Figure S5A). In contrast, lung IL-1β protein and gene expression and TNF-*α* levels did not differ significantly between groups (Figure S5B-D). These results suggest that dietary tryptophan enrichment attenuates pulmonary cytokine responsiveness in a standardized LPS-driven inflammatory challenge.

### A Trp-Rich diet reprograms murine AM responses in an *ex vivo* nutritional IR model

Because our previous work identified AMs as key regulators of lung IR injury, we next focused on AM responses in an *ex vivo* setting.^8^ To determine whether dietary tryptophan enrichment reprograms AMs *in vivo*, we isolated primary murine AMs from Trp-Std and Trp-Rich mice after 2 weeks of dietary intervention and subjected them to an *ex vivo* nutritional IR model designed to mimic IR-associated stress by transiently limiting nutrient and serum availability while maintaining normoxic culture conditions (Figure 4D).^21^ ^,^ ^28^ AMs from Trp-Rich mice released less IL-1β and IL-6 into the supernatant than AMs from Trp-Std mice, indicating that dietary intervention was sufficient to confer a less inflammatory macrophage phenotype *ex vivo* (Figure 4E, F). These findings support the concept that Trp-Rich diet reprograms AM responses *in vivo* and provide an intermediate bridge between the whole-animal phenotype and the downstream *ex vivo* and *in vitro* mechanistic analyses.

### Anti-inflammatory effect of IPA in nutritional IR model with human AMs

Human primary AMs from Donors 1 and 2 were subjected to a nutritional IR model (Figure 4G).^21^ ^,^ ^28^ Based on characteristic autofluorescence, >  90% of the recovered adherent cells were confirmed to be AMs (Figure S6A). Under these conditions, IPA suppressed IL-1β production across 0.01, 0.1, and 1 mM concentrations, and the overall observed effect was consistent across both donors (Figures 4H and S6B, C).

### Comparison of the anti-inflammatory effects of tryptophan metabolites in LPS-stimulated AMs

**Figure 5.**
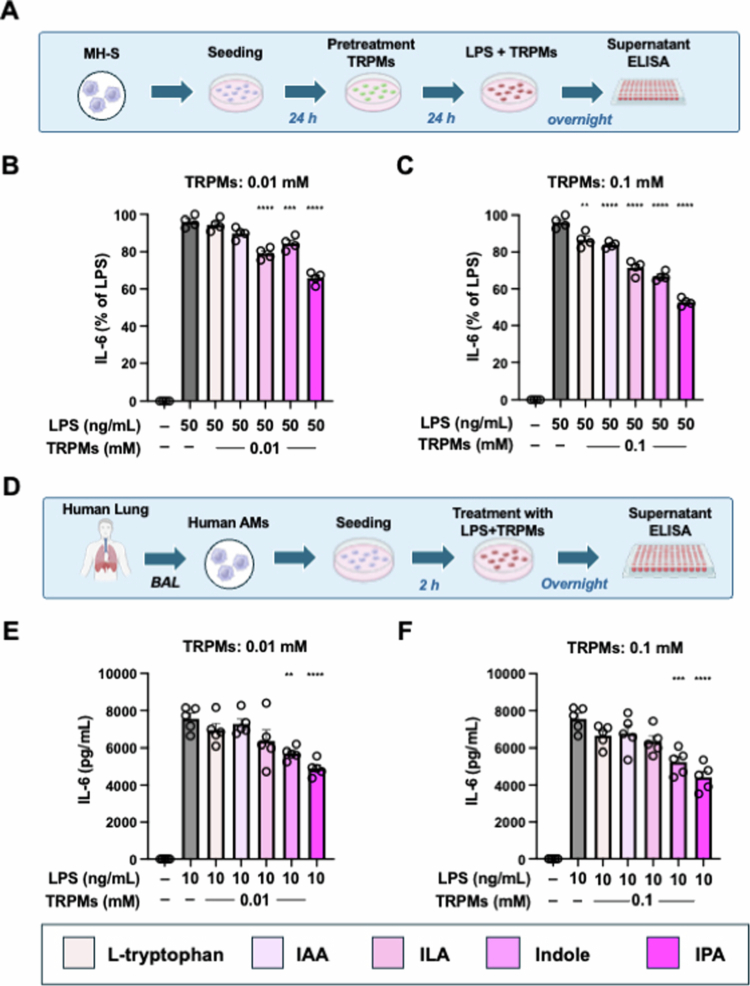
Indole-3-propionate shows the strongest anti-inflammatory effect among representative tryptophan metabolites in murine and human alveolar macrophages. (A) Experimental design for comparing the anti-inflammatory effects of representative indole metabolites, including L-tryptophan, indole-3-acetate (IAA), indole-3-lactate (ILA), indole, and indole-3-propionate (IPA), in MH-S cells stimulated with lipopolysaccharide (LPS). (B, C) At 0.01 and 0.1  mM, IPA produced the greatest suppression of IL-6 among the metabolites tested in LPS-stimulated MH-S cells. (D) Experimental design for comparing representative indole metabolites in primary human alveolar macrophages (AMs) stimulated with LPS. (E, F) IPA showed the strongest suppression of IL-6 in LPS-stimulated primary human AMs at 0.01 and 0.1 mM. Data were analyzed by one-way ANOVA with Tukey’s post hoc test. **: *p* < 0.01, ***: *p* < 0.001, and ****: *p* < 0.0001 vs LPS alone.

Given evidence that AhR signaling can regulate macrophage inflammatory responses to LPS, we next used an LPS-driven inflammatory stimulation model in AMs to compare the anti-inflammatory effects of representative tryptophan-related metabolites.^8^ ^,^ ^42^ Several tryptophan-related metabolites have been reported to exert anti-inflammatory effects, including L-tryptophan, IAA, ILA, indole, and IPA.^43-47^ We therefore compared these five metabolites side-by-side. In MH-S cells, pretreatment with each metabolite followed by overnight co-treatment with LPS was performed (Figure 5A). At 0.01  mM, ILA, indole, and IPA significantly reduced IL-6 after LPS stimulation (Figure 5B). At 0.1  mM, all five metabolites reduced IL-6 (Figure 5C). Across both concentrations, IPA produced the largest reduction in IL-6 among the metabolites tested. We then evaluated the same panel in primary human AMs (Donor 3) using overnight co-treatment with LPS and each metabolite (Figure 5D). At 0.01  mM, indole and IPA significantly suppressed IL-6 (Figure 5E). At 0.1  mM, ILA, indole, and IPA reduced IL-6 (Figure 5F). Together, these results identify IPA as the most consistently potent anti-inflammatory metabolite among major tryptophan-related indole derivatives in both murine and human AMs.

### Anti-inflammatory effect of IPA in AMs

**Figure 6.**
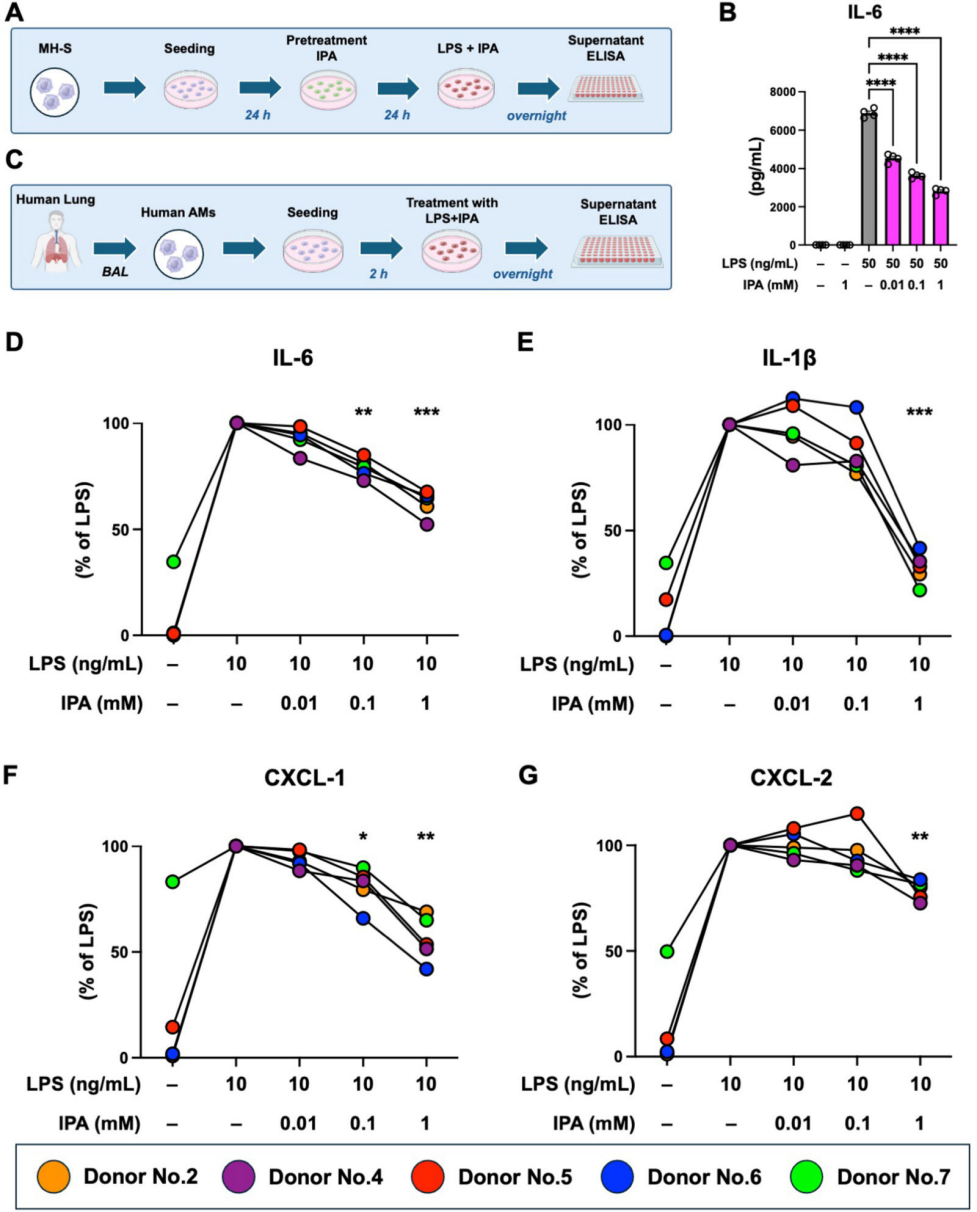
Indole-3-propionate suppresses inflammatory mediator production in murine and human alveolar macrophages in a dose-dependent manner. (A) Experimental design for evaluating the anti-inflammatory effect of indole-3-propionate (IPA) in MH-S cells stimulated with lipopolysaccharide (LPS). (B) IPA reduced LPS-induced IL-6 production in a concentration-dependent manner. (C) Experimental design for evaluating IPA in primary human alveolar macrophages (AMs) stimulated with LPS across five donor lungs. (D–G) IPA dose-dependently attenuated the production of IL-6, IL-1β, CXCL-1, and CXCL-2 in primary human AMs. Baseline cytokine production was detectable in donor No.7. Notably, at 1 mM IPA, IL-1β and CXCL-1 were reduced below baseline in this donor. Detailed data for donor No.7 are shown in Supplemental Figure S8. Statistical analyses were performed using one-way ANOVA with Tukey’s post hoc test in (B) and repeated-measures one-way ANOVA with Dunnett’s post hoc test (C). *: *p* < 0.05, **: *p* < 0.01, and ***: *p* < 0.001.

To define the dose-response relationship for IPA, MH-S cells were treated with IPA (0.01, 0.1, and 1  mM) using pretreatment followed by LPS challenge in the continued presence of IPA (Figure 6A). IPA suppressed LPS-induced inflammation in a concentration-dependent manner, whereas IPA alone did not trigger inflammatory activation (Figure 6B). Similar dose-dependent anti-inflammatory effects were observed for L-tryptophan, IAA, ILA, and indole (Figure S7). We next tested IPA in primary human AMs from five donors (Donors 2 and 4–7) using overnight co-treatment with LPS and IPA (Figure 6C). IPA dose-dependently reduced IL-6, IL-1β, CXCL-1, and CXCL-2 in culture supernatants, consistently across donors (Figures 6D–G). In Donor 7, measurable baseline cytokine production was observed in the absence of LPS, consistent with pre-existing inflammatory activation in the donor lung (Figure S8). Notably, IPA reduced IL-1β and CXCL-1 to below baseline in this donor (Figures S8B-C), supporting the possibility that IPA may retain anti-inflammatory activity even in pre-activated AMs. To explore whether baseline AhR pathway tone influenced donor responsiveness to IPA, we related baseline *AHRR* expression to the magnitude of IPA-mediated IL-6 suppression in primary human AMs. *AHRR* expression was especially higher in Donor No. 4 (Figure S9A). In exploratory donor-level analyses, higher baseline *AHRR* expression was associated with greater IPA-mediated reduction in IL-6 at both 0.01 and 1  mM IPA (Figure S9B, C). These findings are consistent with the possibility that donor-to-donor variation in baseline AhR pathway tone modulates the magnitude of anti-inflammatory responses to IPA. Collectively, these data show that IPA broadly suppresses cytokine and chemokine production in murine and primary human AMs.

#### Pharmacologic AhR blockade attenuates the anti-inflammatory effect of IPA in AMs

**Figure 7.**
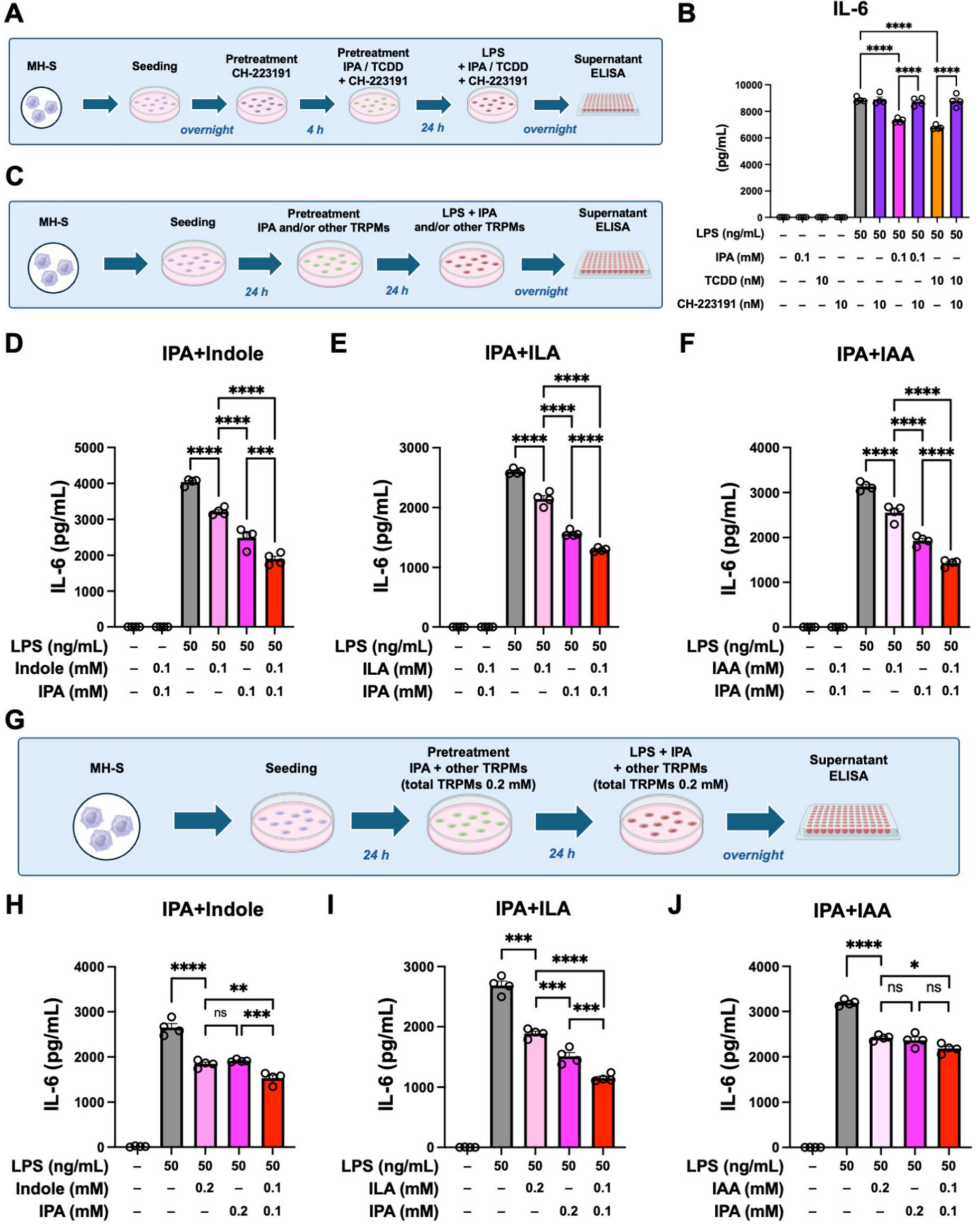
Indole-3-propionate suppresses inflammatory mediator production in association with AhR signaling and exhibits additive and synergistic effects with other indole metabolites. (A) Experimental design to test whether pharmacologic AhR blockade attenuates the anti-inflammatory effect of indole-3-propionate (IPA) in MH-S cells stimulated with lipopolysaccharide (LPS). (B) AhR antagonism with CH-223191 (10 nM) during pre- and co-treatment attenuated the suppressive effect of IPA (0.1  mM) on LPS-induced IL-6 production. The AhR agonist 2,3,7,8-tetrachlorodibenzo-*p*-dioxin (TCDD, 10 nM) served as a positive control. Data for IPA at 0.01 mM are shown in Supplemental Figure S10A, B. (C) Experimental design to evaluate the additive effect of IPA with other indole metabolites in LPS-stimulated MH-S cells. (D–F) Co-treatment with IPA and indole, indole-3-lactate (ILA), and indole-3-acetate (IAA) produced greater suppression of LPS-induced IL-6 than either metabolite alone at 0.1  mM. Data at 0.01 and 1  mM are provided in Supplemental Figure S10D–I. (G) Experimental design to investigate the synergistic effect using a fixed total metabolite concentration in LPS-stimulated MH-S cells. (H–J) At 0.2  mM fixed total concentration, combinations of IPA with indole or ILA suppressed LPS-induced IL-6 more than the corresponding single-metabolite conditions, consistent with synergistic interactions, whereas the IPA + IAA combination did not show a statistically clear synergistic effect. Statistical analysis was performed using one-way ANOVA with Tukey’s post hoc test. *: *p* < 0.05, **: *p* < 0.01, ***: *p* < 0.001, ****: *p* < 0.0001.

Because AhR is a major host receptor for tryptophan metabolites, we tested whether IPA’s anti-inflammatory effects in AMs required AhR signaling. MH-S cells were treated with the AhR antagonist CH-223191, and TCDD was included as a positive-control AhR agonist (Figure 7A). IPA (0.1 mM) and TCDD (10 nM) each suppressed LPS-induced IL-6 production, and these effects were attenuated by CH-223191 (Figure 7B). AhR blockade similarly attenuated IPA-mediated suppression at 0.01  mM, as reflected by IL-6 and TNF-*α* (Figures S10A-B). We also examined *Pparγ*, a transcription factor linked to anti-inflammatory programs and homeostatic differentiation in tissue-resident AMs.^48^ ^,^ ^49^ LPS reduced *Pparγ* expression, whereas IPA restored *Pparγ* toward baseline (Figure S10C). Together, these findings support involvement of AhR signaling in IPA-mediated suppression of inflammatory responses in AMs and suggest concomitant transcriptional shifts consistent with a more immunoregulatory macrophage state.

### Combined effects of IPA with other tryptophan metabolites

Because IPA naturally coexists with other indole derivatives *in vivo*, we examined whether combining IPA with other tryptophan-related metabolites enhances anti-inflammatory efficacy. In MH-S cells, co-treatment with IPA plus indole, ILA, or IAA produced greater suppression of LPS-induced IL-6 than either IPA or the paired metabolite alone at 0.1  mM (Figures 7C, schema, and 7D–F). Similar additive suppression was observed at 0.01 and 1 mM (Figure S10D–I). To probe potential synergy under a fixed total metabolite exposure, we held the total concentration constant at 0.2  mM, comparing a single metabolite at 0.2  mM with a combination of IPA (0.1  mM) plus a second metabolite (0.1  mM) (Figure 7G). Under these fixed total-concentration conditions, combinations of IPA with indole or ILA suppressed IL-6 more strongly than the corresponding single-metabolite conditions (Figure 7H, I), consistent with synergistic interactions, whereas the IPA + IAA combination did not show a statistically clear synergistic effect (Figure 7J). Overall, these results indicate that IPA can act additively with other tryptophan-derived indoles to dampen AM inflammatory responses.

## Discussion

We investigated whether tryptophan enrichment protects against sterile lung IR injury through the gut–lung axis. A two-week dietary intervention reduced lung inflammation after IR injury and improved oxygenation. *Cyp1a1* and *Cyp1b1* expression increased in lung tissue, suggesting enhanced pulmonary AhR signaling. Targeted metabolomics in feces, PV plasma, and lung tissue revealed a coordinated shift in tryptophan metabolism toward the indole branch, with consistent increases in IPA and decreases in kynurenine/IPA ratios across matrices. In mechanistic studies, IPA showed the strongest anti-inflammatory activity among key tryptophan-derived indoles in MH-S cells and primary human AMs, suppressing multiple cytokines and chemokines in a dose-dependent manner as well as in an *ex vivo* nutritional IR model. Pharmacologic AhR blockade attenuated IPA-mediated suppression of inflammatory mediator production, supporting involvement of AhR signaling in this response. Together, these findings are consistent with a model in which diet-associated increases in IPA may reduce AM inflammatory responsiveness in the setting of lung IR injury. However, because we did not directly assess NF-κB pathway activation, these data should be interpreted as evidence of reduced LPS-induced inflammatory mediator production rather than direct suppression of NF-κB activity. As a complementary *in vivo* inflammatory challenge, Trp-Rich feeding also reduced LPS-induced IL-6 responses in both lung tissue and systemic plasma, although this PAMP-driven model should not be interpreted as a direct surrogate for sterile DAMP-driven IR injury.

A Trp-Rich diet reshaped both the gut microbiota composition and the tryptophan metabolite landscape, coinciding with increased IPA in feces, PV plasma, and lung tissue. This rise in IPA is biologically plausible because IPA is widely described as a microbiota-derived tryptophan metabolite, and gut microbial tryptophan metabolites can engage host AhR-associated programs that support barrier homeostasis and immunoregulatory function.^22^ ^,^ ^50^ Notably, *Lactobacillus* and *Bifidobacterium* expanded in the Trp-Rich group, and these taxa have been linked to indole-related metabolism and modulation of host immune tone.^37^ ^,^ ^38^ ^,^ ^51^ ^,^ ^52^ In addition, prior studies using defined microbial systems have shown that IPA-producing bacteria, including *Clostridium sporogenes*, can regulate host IPA availability and inflammatory phenotypes.^53^ ^,^ ^54^ Accordingly, our integrated analyses linked higher IPA levels to lung inflammatory readouts and AhR-related responses. However, we acknowledge that these data are associative and do not directly prove that the increased IPA in our model was microbiota-derived. Host metabolic pathways may also contribute to tryptophan-derived metabolite pools. Therefore, we interpret the microbiome and metabolomics findings as being consistent with, rather than definitive proof of, enhanced microbial tryptophan metabolism along the gut–lung axis.

Building on our observations of multi-compartmental increases in IPA along the gut-lung axis, we next investigated whether IPA could directly reprogram AM inflammatory responses in a manner consistent with our *in vivo* dietary intervention observations. As a complementary standardized inflammatory challenge, the intratracheal LPS experiment showed that Trp-Rich feeding reduced IL-6 responses *in vivo*, supporting the broader concept that the dietary intervention can dampen pulmonary inflammatory responsiveness. Across both MH-S cells and primary human AMs, IPA showed the most potent suppression of LPS-induced cytokine and chemokine production among the indole derivatives tested, with clear dose dependence across physiologic-to-pharmacologic concentration ranges. These results align with prior work showing that AhR signaling can regulate LPS-induced inflammatory responses in macrophages and that IPA can partially activate AhR-related macrophage programs.^23^ ^,^ ^42^ In our study, IPA reduced LPS-induced cytokine and chemokine production in MH-S cells and primary human AMs. However, because we did not directly measure p65 nuclear translocation, IκB degradation, or NF-κB reporter activity, we interpret these findings as reduced inflammatory mediator production rather than direct evidence of NF-κB pathway inhibition. Mechanistically, pharmacologic AhR blockade with CH-223191 attenuated the anti-inflammatory effect of IPA, while the AhR agonist TCDD recapitulated suppression of IL-6. CH-223191 is widely used as a pharmacologic AhR antagonist, although it is ligand-selective and therefore cannot by itself establish complete pathway dependence. Together with prior evidence that AhR signaling negatively regulates LPS-induced inflammatory responses in macrophages and that IPA can partially activate AhR-dependent macrophage programs, our findings support the involvement of AhR signaling in IPA-mediated suppression of AM inflammatory responses.^23^ ^,^ ^53^ ^,^ ^55^ However, because we did not directly assess IPA-induced AhR nuclear translocation in AMs or perform genetic AhR perturbation, these data should be interpreted as evidence supporting AhR involvement rather than definitive proof of complete AhR dependence. Moreover, IPA restored *Pparγ* expression in LPS-stimulated MH-S cells, consistent with established roles for PPARγ in AM differentiation and homeostatic programs.^49^ Since IPA coexists with other indole derivatives *in vivo*, our observation that combinations of IPA with indole or ILA further enhanced suppression of IL-6 is conceptually consistent with the literature showing that distinct microbiota-derived indoles can converge on host immunoregulatory pathways.^56^ ^,^ ^57^

From a translational perspective, our data support the concept that perioperative “dietary prehabilitation” targeting tryptophan metabolism and IPA availability could be a feasible way to modulate lung inflammatory responses, given that IR risk is often predictable in transplantation and cardiothoracic surgery. Consistent with the airway relevance of this metabolic axis, reduced microbiota-derived IPA has been linked to worse allergic airway inflammation *in vivo*, supporting the idea that IPA availability can shape lung immune tone.^47^ Additionally, tryptophan supplementation attenuated LPS-induced acute lung injury in rats, providing additional rationale for leveraging dietary tryptophan availability to modulate lung inflammation.^43^ Beyond diet alone, a more controllable strategy may be microbiome-directed therapy that directly elevates endogenous IPA. In clinical contexts with predictable inflammatory risk, disruption of gut microbial IPA production has been associated with worse postoperative outcomes.^54^ Restoring IPA signaling conferred protective effects, whereas colonization with an IPA-deficient *Clostridium sporogenes* strain abrogated protection.^54^ Complementing this, *Clostridium sporogenes*-derived metabolites (including IPA) have been shown to protect against colonic inflammation, supporting the feasibility of leveraging defined IPA-producing strains as next-generation probiotics.^53^ Synbiotic design may further optimize this approach, as pairing *Clostridium sporogenes* with a matching dietary substrate increased gut-derived IPA levels and improved disease phenotypes *in vivo.* ^58^ More broadly, dietary fiber composition can redirect community tryptophan metabolism toward higher IPA production via microbial interactions, suggesting that diet composition and strain selection should be co-designed rather than considered separately.^59^ Finally, this gut–lung axis model offers practical pharmacodynamic readouts, such as circulating IPA and the kynurenine/IPA ratio, to assess whether interventions engage the intended gut-to-lung metabolic axis in humans.

Several limitations should be considered. First, our *in vivo* study was powered to detect diet-associated differences but used a modest sample size and ultimately focused on male mice because the protective phenotype was not observed in females. Sex hormones can modulate lung IR biology, and future sex-stratified studies will be necessary.^34^ Second, while targeted metabolomics showed reductions in the kynurenine/IPA and kynurenine/tryptophan ratios, these ratios should be interpreted as indirect surrogates of pathway balance and immune-linked tryptophan catabolism rather than direct measures of metabolic flux. Complementary approaches that quantify kinetics or pathway activity in relevant compartments will strengthen mechanistic interpretation.^60^ ^,^ ^61^ Relatedly, although the increase in IPA across feces, PV plasma, and lung tissue was accompanied by gut microbiota remodeling and was consistent with prior literature identifying IPA as a microbiota-derived tryptophan metabolite, our study did not directly establish microbial dependence. We did not use germ-free mice, broad-spectrum antibiotic depletion, isotope tracing, or colonization with defined IPA-producing or IPA-deficient bacteria. Therefore, we cannot exclude contributions from host tryptophan metabolism or determine the relative contribution of microbial versus host pathways to the observed IPA pool. Because diets were administered at the cage level and mice were group-housed by diet, we cannot exclude cage effects in the microbiome analysis. Future studies should include cage-level replication and statistical approaches that account for the cage as an experimental unit. Third, our *in vitro* and *ex vivo* experiments intentionally spanned putative physiologic-to-pharmacologic concentrations of IPA, but the practical exposure of alveolar lining fluid and tissue-resident AMs to tryptophan metabolites *in vivo* remains uncertain, motivating future pharmacokinetic and dosing studies. Fourth, the intratracheal LPS experiment should be interpreted as a standardized PAMP-driven inflammatory challenge rather than a direct model of sterile lung IR injury. Endogenous DAMPs generated during IR injury and exogenous LPS may activate partially overlapping inflammatory pathways, but they are not equivalent stimuli. Therefore, the LPS data support reduced inflammatory responsiveness with Trp-Rich feeding, but do not establish that the same mechanism operates during DAMP-driven sterile IR injury. Fifth, although IPA suppressed LPS-induced cytokine and chemokine production in AMs, we did not directly assess canonical NF-κB activation using p65 nuclear translocation, IκB degradation, or NF-κB reporter assay. Therefore, future studies will be needed to determine whether IPA directly modulates NF-κB activation kinetics in AMs and whether this effect is AhR-dependent. Finally, although pharmacologic AhR antagonism with CH-223191 and the use of TCDD as a positive-control AhR agonist support involvement of AhR signaling in IPA-mediated suppression of AM inflammatory responses, this approach has important limitations. CH-223191 is a useful pharmacologic AhR antagonist but exhibits ligand-selective properties; therefore, antagonist-based experiments cannot substitute for direct assessment of IPA-induced AhR nuclear translocation or genetic AhR loss-of-function approaches. Future studies using AhR immunofluorescence or nuclear fractionation, AhR knockdown or AhR-deficient macrophages, and myeloid- or AM-targeted AhR perturbation *in vivo* will be needed to define the extent to which IPA-mediated protection requires AhR signaling.^62^

## Conclusions

A Trp-Rich diet attenuated sterile lung IR injury, coinciding with gut microbiota remodeling, a systemic and pulmonary shift in tryptophan metabolism toward indole production, and increased endogenous IPA levels across feces, PV plasma, and lung tissue. Consistent with lower lung immune tone, the Trp-Rich diet also dampened LPS-induced IL-6 responses *in vivo*. IPA showed robust anti-inflammatory activity in murine and human AMs, partly associated with AhR signaling, and was enhanced by combining it with other indole metabolites. Together, these findings support further investigation of dietary or microbiome-informed strategies that modulate tryptophan metabolism, increase IPA availability, and reprogram macrophage responses to suppress acute lung IR inflammation.

## Supplementary Material

Supplemental Figure Legend ver6.docx

Supplemental Methods.docx Supplemental Methods.docx

Supplemental Tables.docx Supplemental Tables.docx

## Data Availability

The datasets generated during and/or analyzed during the current study are available from the corresponding author on reasonable request.

## Abbreviations

IR: ischemia-reperfusion
IPA: indole-3-propionate
IAA: indole-3-acetate
ILA: indole-3-lactate
AM: alveolar macrophage
AhR: aryl hydrocarbon receptor

## References

[1] Talaie T , DiChiacchio L , Prasad NK , Pasrija C , Julliard W , Kaczorowski DJ , Zhao Y , Lau CL . Ischemia-reperfusion injury in the transplanted lung: a literature review. Transplant Direct. 2021;7:e652. doi: 10.1097/TXD.0000000000001104. 33437867 PMC7793349

[2] Wang Z , Zhan R , Mo L , Zhang Y , Chen Y , Liu L , Bai Y . Cardiopulmonary bypass-mediated lung ischemia-reperfusion injury: a protective role of sitaxentan. J Int Med Res. 2025;53:3000605251383318. doi: 10.1177/03000605251383318. 41067859 PMC12515309

[3] Laubach VE , Sharma AK . Mechanisms of lung ischemia-reperfusion injury. Curr Opin Organ Transplant. 2016;21:246–252. doi: 10.1097/MOT.0000000000000304. 26945320 PMC4861054

[4] Ta HQ , Kuppusamy M , Sonkusare SK , Roeser ME , Laubach VE . The endothelium: gatekeeper to lung ischemia-reperfusion injury. Respir Res. 2024;25:172. doi: 10.1186/s12931-024-02776-4. 38637760 PMC11027545

[5] Wu W , Meng F , Zhang H , Tian H , Zhang X . Neutrophil PPIF exacerbates lung ischemia-reperfusion injury after lung transplantation by promoting calcium overload-induced neutrophil extracellular traps formation. Int Immunopharmacol. 2024;142:113051. doi: 10.1016/j.intimp.2024.113051. 39236457

[6] Nakagiri T , Köhler NR , Janciauskiene S , Neubert L , Knöfel AK , Pradhan P , Ruhparwar A , Ius F , Immenschuh S . Hemopexin alleviates sterile inflammation in ischemia-reperfusion-induced lung injury. Front Immunol. 2024;15:1451577. doi: 10.3389/fimmu.2024.1451577. 39430764 PMC11487521

[7] Liao F , Scozzi D , Zhou D , Maksimos M , Diedrich C , Cano M , Tague LK , Liu Z , Haspel JA , Leonard JM , et al. Nanoparticle targeting of neutrophil glycolysis prevents lung ischemia-reperfusion injury. Am J Transplant. 2024;24:1382–1394. doi: 10.1016/j.ajt.2024.03.028. 38522826 PMC11305958

[8] Prakash A , Mesa KR , Wilhelmsen K , Xu F , Dodd-o JM , Hellman J . Alveolar macrophages and toll-like receptor 4 mediate ventilated lung ischemia reperfusion injury in mice. Anesthesiology. 2012;117:822–835. doi: 10.1097/ALN.0b013e31826a4ae3. 22890118 PMC3477877

[9] Xu KY , Wu CY , Tong S , Xiong P , Wang SH . The selective Nlrp3 inflammasome inhibitor Mcc950 attenuates lung ischemia-reperfusion injury. Biochem Biophys Res Commun. 2018;503:3031–3037. doi: 10.1016/j.bbrc.2018.08.089. 30146255

[10] Wang Z , Zhang A , Meng W , Wang T , Li D , Liu Z , Liu H . Ozone protects the rat lung from ischemia-reperfusion injury by attenuating NLRP3-mediated inflammation, enhancing Nrf2 antioxidant activity and inhibiting apoptosis. Eur J Pharmacol. 2018;835:82–93. doi: 10.1016/j.ejphar.2018.07.059. 30075224

[11] Jin T , Ai F , Zhou J , Kong L , Xiong Z , Wang D , Lu R , Chen Z , Zhang M . Emodin alleviates lung ischemia-reperfusion injury by suppressing gasdermin D-mediated pyroptosis in rats. Clin Respir J. 2023;17:241–250. doi: 10.1111/crj.13582. 36751097 PMC9978909

[12] Ziaka M , Exadaktylos A . Gut-derived immune cells and the gut-lung axis in ARDS. Crit Care. 2024;28:220. doi: 10.1186/s13054-024-05006-x. 38965622 PMC11225303

[13] Suswał K , Tomaszewski M , Romaniuk A , Świechowska-Starek P , Zygmunt W , Styczeń A , Romaniuk-Suswał M . Gut-lung axis in focus: deciphering the impact of gut microbiota on pulmonary arterial hypertension. J Pers Med. 2023;14:8. doi: 10.3390/jpm14010008. 38276223 PMC10817474

[14] Ashique S , De Rubis G , Sirohi E , Mishra N , Rihan M , Garg A , Reyes RJ , Manandhar B , Bhatt S , Jha NK , et al. Short chain fatty acids: fundamental mediators of the gut-lung axis and their involvement in pulmonary diseases. Chem Biol Interact. 2022;368:110231. doi: 10.1016/j.cbi.2022.110231. 36288778

[15] Eladham MW , Selvakumar B , Saheb Sharif-Askari N , Saheb Sharif-Askari F , Ibrahim SM , Halwani R . Unraveling the gut-lung axis: exploring complex mechanisms in disease interplay. Heliyon. 2024;10:e24032. doi: 10.1016/j.heliyon.2024.e24032. 38268584 PMC10806295

[16] Ye F , Li L , Wang J , Yang H . Advances in gut-lung axis research: clinical perspectives on pneumonia prevention and treatment. Front Immunol. 2025;16:1576141. doi: 10.3389/fimmu.2025.1576141. 40330490 PMC12052896

[17] Kim S , Seo SU , Kweon MN . Gut microbiota-derived metabolites tune host homeostasis fate. Semin Immunopathol. 2024;46:2. doi: 10.1007/s00281-024-01012-x. 38990345 PMC11239740

[18] Zhang D , Jian YP , Zhang YN , Li Y , Gu LT , Sun HH , Liu MD , Zhou HL , Wang YS , Xu ZX . Short-chain fatty acids in diseases. Cell Commun Signal. 2023;21:212. doi: 10.1186/s12964-023-01219-9. 37596634 PMC10436623

[19] Rothhammer V , Mascanfroni ID , Bunse L , Takenaka MC , Kenison JE , Mayo L , Chao CC , Patel B , Yan R , Blain M , et al. Type I interferons and microbial metabolites of tryptophan modulate astrocyte activity and central nervous system inflammation via the aryl hydrocarbon receptor. Nat Med. 2016;22:586–597. doi: 10.1038/nm.4106. 27158906 PMC4899206

[20] Gutiérrez-Vázquez C , Quintana FJ . Regulation of the immune response by the aryl hydrocarbon receptor. Immunity. 2018;48:19–33. doi: 10.1016/j.immuni.2017.12.012. 29343438 PMC5777317

[21] Maruyama D , Tian X , Doan TNM , Liao WI , Chaki T , Taenaka H , Maishan M , Matthay MA , Prakash A . Gut microbiome-derived propionate reprograms alveolar macrophages metabolically and regulates lung injury responses in mice. Gut Microbes. 2025;18:2606486. doi: 10.1080/19490976.2025.2606486. 41467904 PMC12758369

[22] Konopelski P , Mogilnicka I . Biological effects of Indole-3-Propionic acid, a gut microbiota-derived metabolite, and its precursor tryptophan in Mammals' health and disease. Int J Mol Sci. 2022;23:1222. doi: 10.3390/ijms23031222. 35163143 PMC8835432

[23] Huang ZB , Hu Z , Lu CX , Luo SD , Chen Y , Zhou ZP , Hu JJ , Zhang FL , Deng F , Liu KX . Gut microbiota-derived indole 3-propionic acid partially activates aryl hydrocarbon receptor to promote macrophage phagocytosis and attenuate septic injury. Front Cell Infect Microbiol. 2022;12:1015386. doi: 10.3389/fcimb.2022.1015386. 36299625 PMC9589056

[24] Han Z , Fu J , Gong A , Ren W . Bacterial indole-3-propionic acid inhibits macrophage IL-1β production through targeting methionine metabolism. Sci China Life Sci. 2025;68:1118–1131. doi: 10.1007/s11427-024-2789-1. 39825207

[25] Ren T , Li D , Sun F , Pan L , Wang A , Li X , Bao Y , Zhang M , Zheng F , Yue H . Indole propionic acid regulates gut immunity: mechanisms of metabolite-driven immunomodulation and barrier integrity. J Microbiol Biotechnol. 2025;35:e2503045. doi: 10.4014/jmb.2503.03045. 40825672 PMC12375546

[26] Zelante T , Iannitti RG , Cunha C , De Luca A , Giovannini G , Pieraccini G , Zecchi R , D'Angelo C , Massi-Benedetti C , Fallarino F , et al. Tryptophan catabolites from microbiota engage aryl hydrocarbon receptor and balance mucosal reactivity via interleukin-22. Immunity. 2013;39:372–385. doi: 10.1016/j.immuni.2013.08.003. 23973224

[27] Kumar A , Patel VS , Harding JN , You D , Cormier SA . Exposure to combustion derived particulate matter exacerbates influenza infection in neonatal mice by inhibiting IL22 production. Part Fibre Toxicol. 2021;18:43. doi: 10.1186/s12989-021-00438-7. 34906172 PMC8670221

[28] Maruyama D , Doan TNM , Tian X , Chaki T , Yeh JJ , Layden BT , Prakash A . Gut microbiota-derived lipopolysaccharides and short-chain fatty acids regulate immune responses via FFAR2/FFAR3 in lung ischemia-reperfusion injury. Ann N Y Acad Sci. 2025;1553:449–460. doi: 10.1111/nyas.70043. 41031863 PMC12987534

[29] Liao WI , Maruyama D , Kianian F , Tat C , Tian X , Hellman J , Dodd OJ , Prakash A . A mouse model of orotracheal intubation and ventilated lung ischemia reperfusion surgery. J Vis Exp. 2022;187:64383. doi: 10.3791/64383. PMC9752345 36155626

[30] Lu Y , Zhou G , Ewald J , Pang Z , Shiri T , Xia J . MicrobiomeAnalyst 2.0: comprehensive statistical, functional and integrative analysis of microbiome data. Nucleic Acids Res. 2023;51:W310–8. doi: 10.1093/nar/gkad407. 37166960 PMC10320150

[31] Chong J , Liu P , Zhou G , Xia J . Using MicrobiomeAnalyst for comprehensive statistical, functional, and meta-analysis of microbiome data. Nat Protoc. 2020;15:799–821. doi: 10.1038/s41596-019-0264-1. 31942082

[32] Pang Z , Lu Y , Zhou G , Hui F , Xu L , Viau C , Spigelman AF , MacDonald PE , Wishart DS , Li S , et al. MetaboAnalyst 6.0: towards a unified platform for metabolomics data processing, analysis and interpretation. Nucleic Acids Res. 2024;52:W398–406. doi: 10.1093/nar/gkae253. 38587201 PMC11223798

[33] Ewald JD , Zhou G , Lu Y , Kolic J , Ellis C , Johnson JD , Macdonald PE , Xia J . Web-based multi-omics integration using the analyst software suite. Nat Protoc. 2024;19:1467–1497. doi: 10.1038/s41596-023-00950-4. 38355833

[34] Dai P , He J , Wei Y , Xu M , Zhao J , Zhou X , Tang H . High dose of estrogen protects the lungs from ischemia-reperfusion injury by downregulating the angiotensin II signaling pathway. Inflammation. 2024;47:1248–1261. doi: 10.1007/s10753-024-01973-z. 38386131

[35] Zhang L , Savas U , Alexander DL , Jefcoate CR . Characterization of the mouse Cyp1B1 gene. Identification of an enhancer region that directs aryl hydrocarbon receptor-mediated constitutive and induced expression. J Biol Chem. 1998;273:5174–5183. doi: 10.1074/jbc.273.9.5174. 9478971

[36] Kobayashi A , Sogawa K , Fujii-Kuriyama Y . Cooperative interaction between AhR.Arnt and Sp1 for the drug-inducible expression of CYP1A1 gene. J Biol Chem. 1996;271:12310–12316. doi: 10.1074/jbc.271.21.12310. 8647831

[37] Wang G , Fan Y , Zhang G , Cai S , Ma Y , Yang L , Wang Y , Yu H , Qiao S , Zeng X . Microbiota-derived indoles alleviate intestinal inflammation and modulate microbiome by microbial cross-feeding. Microbiome. 2024;12:59. doi: 10.1186/s40168-024-01750-y. 38504383 PMC10949743

[38] Meng D , Sommella E , Salviati E , Campiglia P , Ganguli K , Djebali K , Zhu W , Walker WA . Indole-3-lactic acid, a metabolite of tryptophan, secreted by bifidobacterium longum subspecies infantis is anti-inflammatory in the immature intestine. Pediatr Res. 2020;88:209–217. doi: 10.1038/s41390-019-0740-x. 31945773 PMC7363505

[39] Badawy AA , Guillemin G . The plasma [Kynurenine]/[Tryptophan] ratio and indoleamine 2,3-Dioxygenase: time for appraisal. Int J Tryptophan Res. 2019;12:1178646919868978. doi: 10.1177/1178646919868978. 31488951 PMC6710706

[40] Ploder M , Spittler A , Kurz K , Neurauter G , Pelinka LE , Roth E , Fuchs D . Accelerated tryptophan degradation predicts poor survival in trauma and sepsis patients. Int J Tryptophan Res. 2010;3:61–67. doi: 10.4137/IJTR.S3983. 22084588 PMC3195245

[41] Dhariwal A , Chong J , Habib S , King IL , Agellon LB , Xia J . MicrobiomeAnalyst: a web-based tool for comprehensive statistical, visual and meta-analysis of microbiome data. Nucleic Acids Res. 2017;45:W180–8. doi: 10.1093/nar/gkx295. 28449106 PMC5570177

[42] Kimura A , Naka T , Nakahama T , Chinen I , Masuda K , Nohara K , Fujii-Kuriyama Y , Kishimoto T . Aryl hydrocarbon receptor in combination with Stat1 regulates LPS-induced inflammatory responses. J Exp Med. 2009;206:2027–2035. doi: 10.1084/jem.20090560. 19703987 PMC2737163

[43] Liu S , She F , Zhang W , Hu X , Zhao X , Yao Y . Tryptophan decreases the intensity of lipopolysaccharide-induced acute lung injury in a rat model. Amino Acids. 2020;52:1139–1147. doi: 10.1007/s00726-020-02878-5. 32789611

[44] Shaheen N , Miao J , Li D , Xia B , Baoyinna B , Zhao Y , Zhao J . Indole-3-Acetic acid protects against lipopolysaccharide-induced endothelial cell dysfunction and lung injury through the activation of USP40. Am J Respir Cell Mol Biol. 2024;71:307–317. doi: 10.1165/rcmb.2024-0159OC. 38761166 PMC11376244

[45] Yu K , Li Q , Sun X , Peng X , Tang Q , Chu H , Zhou L , Wang B , Zhou Z , Deng X , et al. Bacterial indole-3-lactic acid affects epithelium-macrophage crosstalk to regulate intestinal homeostasis. Proc Natl Acad Sci USA. 2023;120:e2309032120. doi: 10.1073/pnas.2309032120. 37903267 PMC10636326

[46] Bansal T , Alaniz RC , Wood TK , Jayaraman A . The bacterial signal indole increases epithelial-cell tight-junction resistance and attenuates indicators of inflammation. Proc Natl Acad Sci USA. 2010;107:228–233. doi: 10.1073/pnas.0906112107. 19966295 PMC2806735

[47] Perdijk O , Butler A , Macowan M , Chatzis R , Bulanda E , Grant RD , Harris NL , Wypych TP , Marsland BJ . Antibiotic-driven dysbiosis in early life disrupts indole-3-propionic acid production and exacerbates allergic airway inflammation in adulthood. Immunity. 2024;57:1939–54.e7. doi: 10.1016/j.immuni.2024.06.010. 39013465

[48] Bazzan E , Turato G , Tinè M , Radu CM , Balestro E , Rigobello C , Biondini D , Schiavon M , Lunardi F , Baraldo S , et al. Dual polarization of human alveolar macrophages progressively increases with smoking and COPD severity. Respir Res. 2017;18:40. doi: 10.1186/s12931-017-0522-0. 28231829 PMC5324331

[49] Schneider C , Nobs SP , Kurrer M , Rehrauer H , Thiele C , Kopf M . Induction of the nuclear receptor PPAR-γ by the cytokine GM-CSF is critical for the differentiation of fetal monocytes into alveolar macrophages. Nat Immunol. 2014;15:1026–1037. doi: 10.1038/ni.3005. 25263125

[50] Scott SA , Fu J , Chang PV . Microbial tryptophan metabolites regulate gut barrier function via the aryl hydrocarbon receptor. Proc Natl Acad Sci USA. 2020;117:19376–19387. doi: 10.1073/pnas.2000047117. 32719140 PMC7431026

[51] Henrick BM , Rodriguez L , Lakshmikanth T , Pou C , Henckel E , Arzoomand A , Olin A , Wang J , Mikes J , Tan Z , et al. Bifidobacteria-mediated immune system imprinting early in life. Cell. 2021;184:3884–3898.e11. doi: 10.1016/j.cell.2021.05.030. 34143954

[52] Cervantes-Barragan L , Chai JN , Tianero MD , Di Luccia B , Ahern PP , Merriman J , Cortez VS , Caparon MG , Donia MS , Gilfillan S , et al. Lactobacillus reuteri induces gut intraepithelial CD4(+)CD8αα(+) T cells. Science. 2017;357:806–810. doi: 10.1126/science.aah5825. 28775213 PMC5687812

[53] Krause FF , Mangold KI , Ruppert AL , Leister H , Hellhund-Zingel A , Lopez Krol A , Pesek J , Watzer B , Winterberg S , Raifer H , et al. Clostridium sporogenes-derived metabolites protect mice against colonic inflammation. Gut Microbes. 2024;16:2412669. doi: 10.1080/19490976.2024.2412669. 39397690 PMC11485882

[54] Zhou X , Wu X , Wu Y , Yang L , Shi E , Ding W , Chen L , Shi X , Feng X , Su C , et al. Indole-3-Propionic acid, a gut microbiota metabolite, protects against the development of postoperative delirium. Ann Surg. 2023;278: e1164−74. doi: 10.1097/SLA.0000000000005886. 37185230 PMC10603211

[55] Zhao B , Degroot DE , Hayashi A , He G , Denison MS . CH223191 is a ligand-selective antagonist of the ah (Dioxin) receptor. Toxicol Sci. 2010;117:393–403. doi: 10.1093/toxsci/kfq217. 20634293 PMC2940411

[56] Sun J , Zhang Y , Kong Y , Ye T , Yu Q , Kumaran Satyanarayanan S , Su KP , Liu J . Microbiota-derived metabolite indoles induced aryl hydrocarbon receptor activation and inhibited neuroinflammation in APP/PS1 mice. Brain Behav Immun. 2022;106:76–88. doi: 10.1016/j.bbi.2022.08.003. 35961580

[57] Peesh P , Blasco-Conesa MP , El Hamamy A , Khan R , Guzman GU , Honarpisheh P , Mohan EC , Goodman GW , Nguyen JN , Banerjee A , et al. Benefits of equilibrium between microbiota- and host-derived ligands of the aryl hydrocarbon receptor after stroke in aged Male mice. Nat Commun. 2025;16:1767. doi: 10.1038/s41467-025-57014-2. 39971928 PMC11839985

[58] Li L , Yang C , Jia M , Wang Y , Zhao Y , Li Q , Gong J , He Y , Xu K , Liu X , et al. Synbiotic therapy with clostridium sporogenes and xylan promotes gut-derived indole-3-propionic acid and improves cognitive impairments in an Alzheimer's disease mouse model. Food Funct. 2024;15:7865–7882. doi: 10.1039/D4FO00886C. 38967039

[59] Sinha AK , Laursen MF , Brinck JE , Rybtke ML , Hjørne AP , Procházková N , Pedersen M , Roager HM , Licht TR . Dietary fibre directs microbial tryptophan metabolism via metabolic interactions in the gut microbiota. Nat Microbiol. 2024;9:1964–1978. doi: 10.1038/s41564-024-01737-3. 38918470 PMC11306097

[60] Jones SP , Franco NF , Varney B , Sundaram G , Brown DA , de Bie J , Lim CK , Guillemin GJ , Brew BJ . Expression of the kynurenine pathway in human peripheral blood mononuclear cells: implications for inflammatory and neurodegenerative disease. PLoS One. 2015;10:e0131389. doi: 10.1371/journal.pone.0131389. 26114426 PMC4482723

[61] O'Connor JC , André C , Wang Y , Lawson MA , Szegedi SS , Lestage J , Castanon N , Kelley KW , Dantzer R . Interferon-gamma and tumor necrosis factor-alpha mediate the upregulation of indoleamine 2,3-dioxygenase and the induction of depressive-like behavior in mice in response to bacillus calmette-guerin. J Neurosci. 2009;29:4200–4209. doi: 10.1523/JNEUROSCI.5032-08.2009. 19339614 PMC2835569

[62] Bessede A , Gargaro M , Pallotta MT , Matino D , Servillo G , Brunacci C , Bicciato S , Mazza EM , Macchiarulo A , Vacca C , et al. Aryl hydrocarbon receptor control of a disease tolerance defence pathway. Nature. 2014;511:184–190. doi: 10.1038/nature13323. 24930766 PMC4098076

